# Direct Gene Transfer with IP-10 Mutant Ameliorates Mouse CVB3-Induced Myocarditis by Blunting Th1 Immune Responses

**DOI:** 10.1371/journal.pone.0018186

**Published:** 2011-03-22

**Authors:** Yan Yue, Jun Gui, Wenqing Ai, Wei Xu, Sidong Xiong

**Affiliations:** 1 Laboratory of Infection and Immunity, Institutes of Biology and Medical Sciences, Soochow University, Suzhou, People's Republic of China; 2 Institute for Immunobiology, Shanghai Medical College, Fudan University, Shanghai, People's Republic of China; Istituto Dermopatico dell'Immacolata, Italy

## Abstract

**Background:**

Myocarditis is an inflammation of the myocardium that often follows the enterovirus infections, with coxsackievirus B3 (CVB3) being the most dominant etiologic agent. We and other groups previously reported that chemokine IP-10 was significantly induced in the heart tissue of CVB3-infected mice and contributed to the migration of massive inflammatory cells into the myocardium, which represents one of the most important mechanisms of viral myocarditis. To evaluate the direct effect of IP-10 on the inflammatory responses in CVB3 myocarditis, herein an IP-10 mutant deprived of chemo-attractant function was introduced into mice to antagonize the endogenous IP-10 activity, and its therapeutic effect on CVB3-induced myocarditis was evaluated.

**Methodology/Principal Findings:**

The depletion mutant pIP-10-AT, with an additional methionine after removal of the 5 N-terminal amino acids, was genetically constructed and intramuscularly injected into BALB/c mice after CVB3 infection. Compared with vector or no treatment, pIP-10-AT treatment had significantly reduced heart/body weight ratio and serum CK-MB level, increased survival rate and improved heart histopathology, suggesting an ameliorated myocarditis. This therapeutic effect was not attributable to an enhanced viral clearance, but to a blunted Th1 immune response, as evidenced by significantly decreased splenic CD4^+^/CD8^+^IFN-γ^+^ T cell percentages and reduced myocardial Th1 cytokine levels.

**Conclusion/Significance:**

Our findings constitute the first preclinical data indicating that interfering *in vivo* IP-10 activity could ameliorate CVB3 induced myocarditis. This strategy may represent as a new therapeutic approach in treating viral myocarditis.

## Introduction

Viral myocarditis represents a leading cause of sudden death in young adults. Up to 20% of patients with histological evidence of myocarditis will ultimately develop dilated cardiomyopathy, a fatal disease leaving heart transplantation as the only treatment [1]–[3]. Enteroviruses of the picornavirus family are considered to be the dominant etiology of viral myocarditis, with coxsackievirus B3 (CVB3) being the most common one. The murine model of CVB3-induced myocarditis has been developed that shares many characteristics with human disease. Despite decades of extensive effort, the pathogenesis of viral myocarditis is still not fully understood. Studies in the murine CVB3 myocarditis model have found that although CVB3 can directly destroy myocardium, the strong host Th1 immune responses may play a more critical pathogenetic role in the course of viral myocarditis, verified by the improvement of heart injury and function by immune modulating and inhibiting agents [4]–[6].

During CVB3 infection, massive myocardial immune cell inflammation could be observed in the mouse model [7]. This process of immune cell trafficking and infiltration is mainly controlled by chemokines and their receptors. It has been proved that MCP-1 [8], MIP-1β and MIP-3β [9] are involved in the cardiac-directed migration of mononuclear cells in viral myocarditis. Beside of their chemo-attractant function, chemokines are also known for their properties to modulate the direction and strength of immune responses including cell activation and differentiation, thus to influence the severity and prognosis of inflammatory heart diseases [10]–[12]. It indicates that chemokines might be a potential target of molecular therapy for viral myocarditis.

Recently, we and other groups reported that expression of CXC chemokine interferon-inducible protein 10 (IP-10) was induced in the heart tissue during CVB3 infection and may play a role in the initiation and progression of myocardial inflammation [13]–[15]. Through its receptor CXCR3, IP-10 robustly attracts activated NK and T cells to infection sites to eliminate invaded pathogens [16], [17], and leads to the remission of pathogen-caused tissue injury. However, as a Th1-type chemokine, IP-10 preferentially promotes Th1 immune responses which have been proved to be detrimental to viral myocarditis [18]. Therefore, we speculate that IP-10 may have dual effects (protective versus immune pathologic) on the CVB3-induced myocarditis. Herein we hypothesize that blocking endogenous IP-10 activity after CVB3 infection may counteract its chemotactic activity and compromise the excessive pathological injury in the heart tissue mediated by viral induced Th1 immune responses.

A mutant IP-10, designated as IP-10-AT, with a methionine added following the removal of the 5 N-terminal amino acids, was genetically constructed. It was designed to antagonize the chemo-attractant effect of endogenous IP-10 by competitively binding to CXCR3 receptor [19]. Injection of pIP-10-AT plasmid via intramuscular route has shown the efficient expression *in vivo* and the obvious blocking effect on IP-10 activity in remote target organs [19]. In the present study, pIP-10-AT was intramuscularly injected into BALB/c mice after CVB3 infection, and its therapeutic efficiency against CVB3-induced myocarditis was evaluated.

## Results

### CVB3 infection up-regulates the cardiac IP-10 expression in a time- and dose-dependent manner

**Figure 1:**
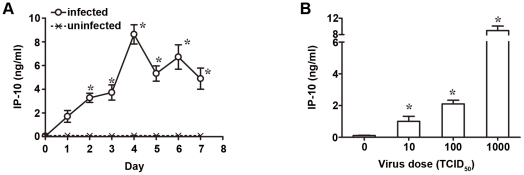
Expression kinetics of IP-10 in the heart tissue following CVB3 infection. (A) BALB/c mice were infected with 10^3^ TCID_50_ dose of CVB3, and the expression level of IP-10 in heart tissue was detected by ELISA assays at different time points. (B) Expression level of IP-10 in heart tissue was detected at day 4 following different doses of CVB3 infection. The results were presented as the mean ±SD of three separate experiments. *, *p*<0.05.

To understand whether IP-10 play a role in CVB3-induced myocarditis, the kinetic expression of IP-10 following CVB3 infection was first observed. BALB/c mice were infected with 10^3^ TCID_50_ dose of CVB3 for various time periods or with different CVB3 doses for 4 days, the heart tissues were collected, homogenized and then subjected to ELISA assays. As shown in Fig. 1A, hardly any cardiac IP-10 could be detected before virus infection. However, after CVB3 infection, slightly up-regulated IP-10 expression was observed as early as 1 day post-infection, and achieved the maximum at day 4, with significantly higher IP-10 level (8.62 ng/ml) than that of day 0 (*p*<0.05). IP-10 expression could be induced by CVB3 at a dose as low as 10 TCID_50_ and correspondingly augmented as CVB3 dose increased (Fig. 1B). These results indicated that IP-10 expression was induced robustly by CVB3 infection in a time- and dose-dependent manner.

### Construction, expression and chemotaxis-antagonizing function of pIP-10-AT

**Figure 2:**
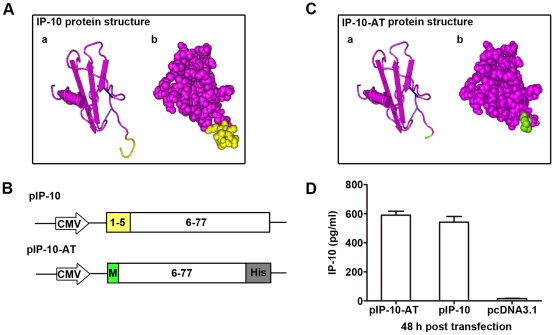
Construction and expression of pIP-10-AT. (A) Structure of IP-10 protein. a, Ribbon diagram of IP-10 monomer (Protein Data Bank number 2R3Z). b, Space filled model of IP-10 in the same orientation as a. Basic residues were highlighted in pink. N-terminal residues 1–5 were critical residues for chemotaxis and highlighted in yellow. (B) Schematic representation of plasmids encoding IP-10 and IP-10-AT. (C) Structure of IP-10-AT protein. a, Ribbon diagram of IP-10-AT. b, Space filled model of IP-10-AT in the same orientation as a. Basic residues lacking the first 5 N-terminal amino acids were highlighted in pink and an additonal methionine was highlighted in green. (D) Expression of pIP-10-AT *in vitro*. 293T cells were transfected with the indicated plasmids, and 48 h later cell supernatant was collected and subjected to ELISA assay. The results were presented as the mean ±SD of three separate experiments.

To antagonize the chemotaxic effect of endogenous IP-10, a mutant IP-10 plasmid designated as pIP-10-AT lacking the 5 N-terminal amino acids was constructed. Native IP-10 protein exhibits the classical chemokine fold, a three-stranded β-sheet overlaid by a α-helix, and is stabilized by disulfide bonds between two pairs of conserved N-terminal cysteines (Fig. 2A). After depleting the chemo-attractant essential part of IP-10 (highlighted in yellow), a methionine and a 6×His tag were respectively added to the N- and C-terminuses of the truncated protein to form the mutant IP-10-AT (Fig. 2B and C).

**Figure 3:**
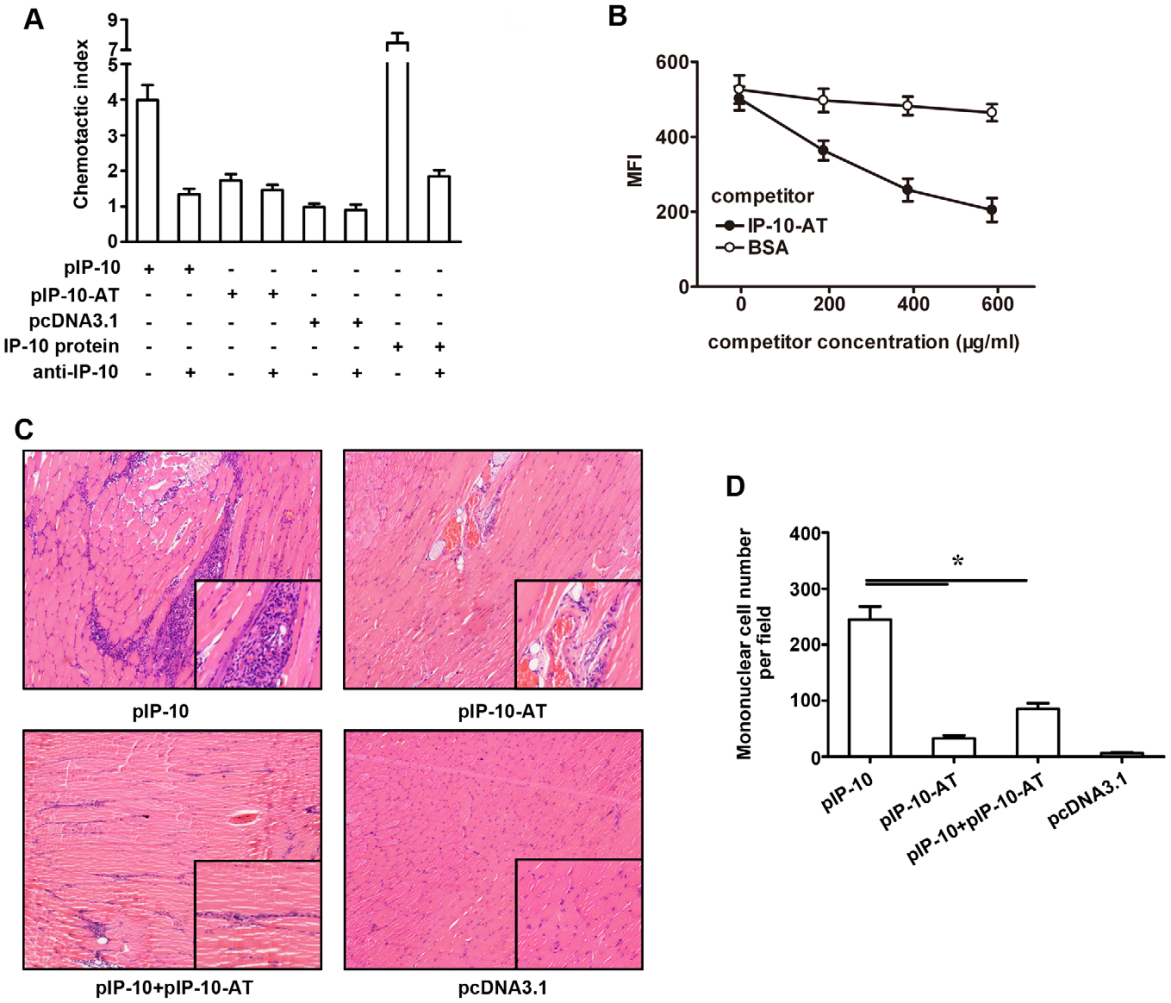
Analysis of the chemotactic and chemotaxis-antagonizing effects of pIP-10-AT. (A) Chemotactic effect of pIP-10-AT *in vitro*. 293T cells were transfected with the indicated plasmids; 48 h later cell supernatant was collected and subjected to chemotaxis assays. (B) Competitive binding assay. CHO/mCXCR3 cells were incubated with FITC-IP-10 protein (20 µg/ml) and unlabeled competitor IP-10-AT protein (200-600 µg/ml). Unlabeled BSA was used as a negative control. Cells were washed extensively to remove unbound protein and analyzed by flow cytometry. Mean fluorescence intensities (MFI) are plotted as a function of increasing quantities of competitor proteins. Error bars represent three independent experiments. (C) Chemotactic and antagnizing effects of pIP-10-AT *in vivo*. Muscular sections at the injection sites were prepared 3 days after pIP-10 or pIP-10-AT intramuscular injection. (D) Recruited cell number in muscular tissues of mice receiving indicated plasmids. The results were presented as the mean ±SD of three separate experiments.

To verify the transcriptional efficiency of plasmids *in vitro*, a lipofectamine-aided transfection assay was conducted on 293T cells, then IP-10-AT and IP-10 expressions in supernatants were analyzed by sandwich ELISA assays. As shown in Fig. 2D, similar expressions of IP-10-AT (590 pg/ml) and IP-10 (540 pg/ml) were detected at 48 h post-transfection. Next, the chemotaxis-antagonizing ability of IP-10-AT was evaluated *in vitro* and *in vivo*. It was found that expressed IP-10-AT protein was deprived of the chemotaxis function towards immune cells *in vitro* (Fig. 3A). And when administrated together, mononuclear cells infiltration in pIP-10 injected muscles could be robustly abrogated by pIP-10-AT co-injection (Fig. 3C and D). These data indicated that IP-10-AT mutant could be efficiently expressed and antagonize the IP-10-mediated immune cell recruitment *in vitro* and *in vivo*. The competitive binding ability of IP-10-AT with IP-10 to the receptor CXCR3 was also detected. As shown in Fig. 3B, a distinct decrease of fluorescence intensity of CHO/mCXCR3 cells was observed when FITC-IP-10 was added with unlabeled IP-10-AT protein. The higher concentration of IP-10-AT, the lower fluorescence intensity was seen, indicating that IP-10-AT competitively inhibited the binding of FITC-IP-10 to CXCR3 on CHO cells. No such shift of fluorescence intensity was observed with the addition of unlabeled BSA.

### Treatment with pIP-10-AT protects mice from CVB3-induced myocarditis

**Figure 4:**
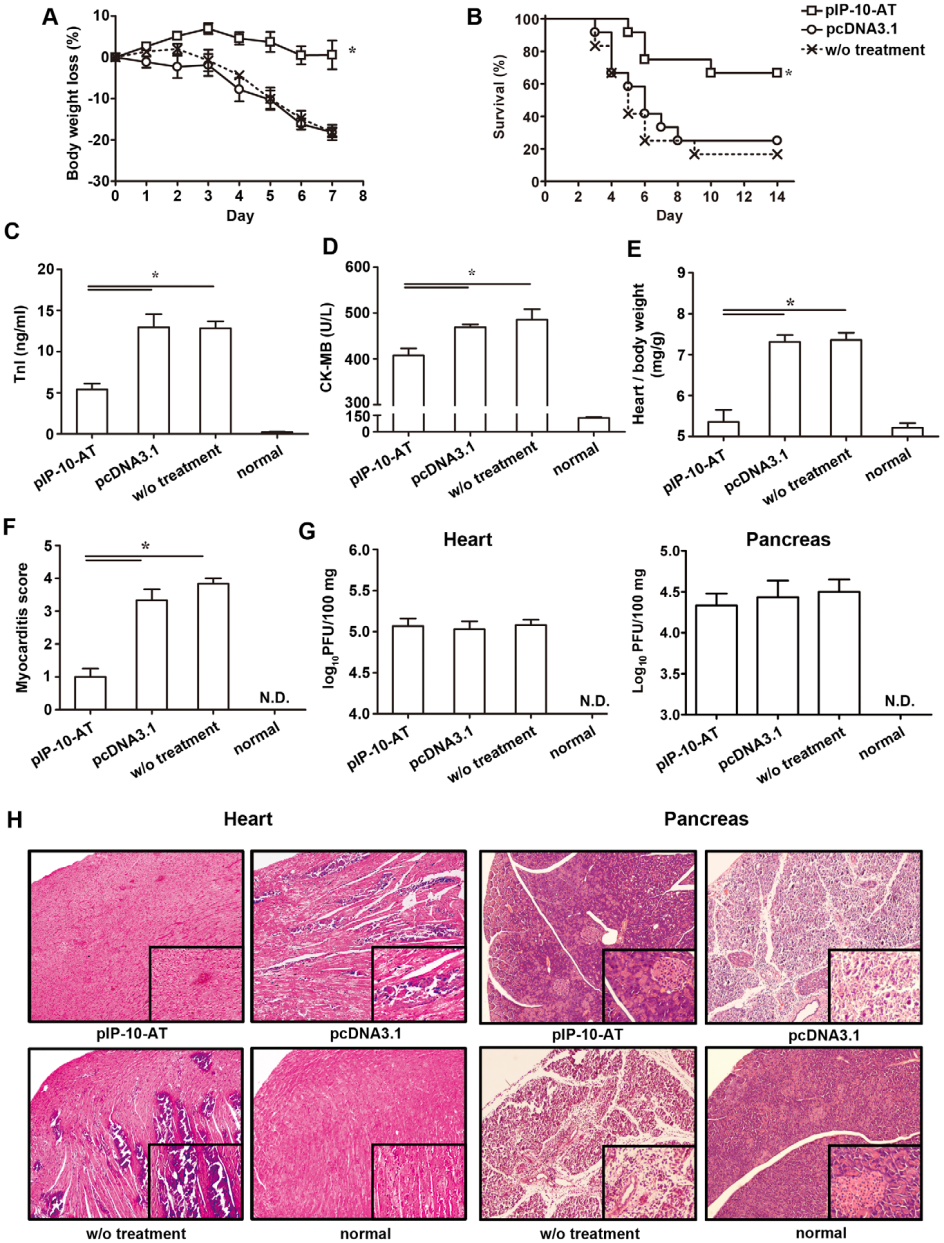
pIP-10-AT administration ameliorates CVB3-induced myocarditis. Mice were infected with 10^3^ TCID_50_ dose of CVB3 and administrated intramuscularly with 2 doses of pIP-10-AT or pcDNA3.1 at day 0 and 3. The body weight change (A) and survival rate (B) were respectively monitored daily until day 7 and day 14 post-infection. Meanwhile, serological indices of myocarditis, the activity of TnI (C) and level of CK-MB (D) in mouse serum were also detected on day 8 post-infection. (E) The ratio of heart weight to body weight was evaluated. (F) Paraffin sections of heart tissues were prepared and cardiac inflammation was revealed by HE staining. The severity of myocarditis was scored by a standard 0–4 scale according to the foci of mononuclear infiltration and myocardial necrosis. *, *p*<0.05. (G) Viral loads in the heart and pancreas tissues on day 8 post-infection were determined by plaque assay on Hela cells. *, *p*<0.05; N.D., not detected. (H) Representative heart and pancreas sections were shown for each group, magnification ×200.

To assess the therapeutic effect of IP-10-AT mutant on CVB3 myocarditis, mice were infected with 10^3^ TCID_50_ CVB3, and then intramuscularly injected with 2 dose of 100 µg pIP-10-AT at day 0 and 3. The survival rate and body weight were monitored. As shown in Fig. 4A and B, mice with no or pcDNA3.1 treatment underwent a dramatic and continuous loss of body weight as maximal to 20%, and more than 70% mice died within 14 days post-infection. On the contrary, mice receiving pIP-10-AT had a little fluctuation in body weight and a significantly improved survival rate (66.7%). Consistent with that, serological indices of myocarditis, TnI and CK-MB, were also significantly improved by pIP-10-AT treatment compared with pcDNA3.1- or non-treated mice (Fig. 4C and D), indicating a significantly reduced myocardial injury.

To see the situation of heart tissue, pIP-10-AT treatment led to a significantly reduced heart/body weight ratio compared with control groups (5.35±0.51 vs. 7.31±0.30, 7.36±0.30 mg/g, *p*<0.05) (Fig. 4E). Significantly improved histopathology of heart tissue, evidenced by fewer inflammation and limited necrosis lesions (Fig. 4F and H), was also observed indicating the ameliorated myocarditis. Consistently, nearly complete destructions of pancreas were showed in control mice on day 8 post-infection, while there was only minimal to mild inflammation or little damage was seen in pIP-10-AT treated mice (Fig. 4H). Theses protective effects were not due to the alteration of cardiac or pancreatic viral load as evidenced by PFU assay (Fig. 4G).

### Reduced Th1 immune responses by pIP-10-AT treatment

**Figure 5:**
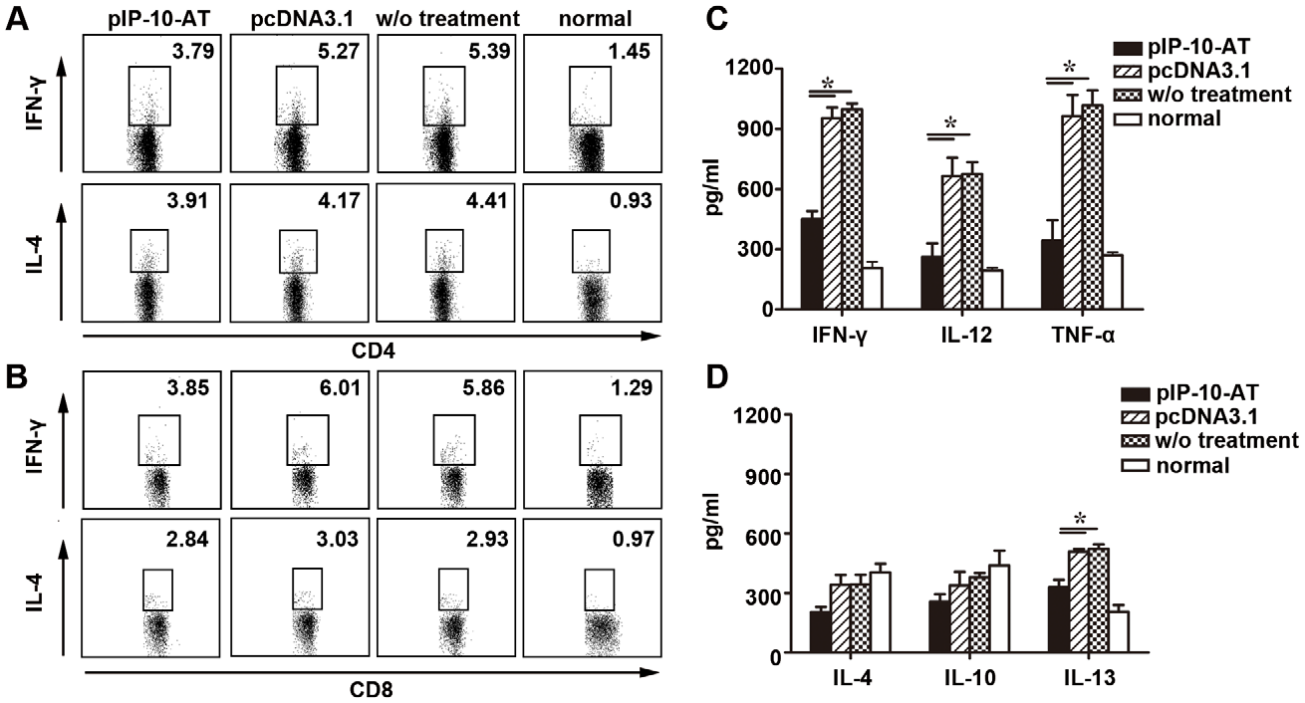
The systemic and local Th1/Th2 immune responses after pIP-10-AT treatment. Mice were infected with 10^3^ TCID_50_ dose of CVB3 and received 2 doses of 100 µg of pIP-10-AT or pcDNA3.1 by intramuscular injection on day 0 and day 3. At day 8, splenocytes (2×10^6^/ml) were isolated and reactivated *in vitro* with 10 µg/ml of plate-bound anti-CD3 and 2 µg/ml soluble anti-CD28 antibody for 24 h. For the final 4 h, 1 µg/ml of GolgiStop was added. Cells were harvested and stained with FITC-conjugated anti-mouse CD4 or anti-mouse CD8. After washing, cells were fixed, permeabilized and then stained with PE-conjugated anti-mouse IFN-γ or anti-mouse IL-4. The percentage of IFN-γ*-* or IL-4-secreting CD4^+^ (A) or CD8^+^ (B) T cells were analyzed by flow cytometry. Meanwhile, the heart tissues were homogenized, the expression levels of Th1 (C) and Th2 (D) cytokines were determined by ELISA assays. Each group contains 6 mice.

CVB3-induced excessive Th1 immune responses were proved to play an important role in the initiation and progression of CVB3 myocarditis. To investigate the immune-modulation mechanism of pIP-10-AT, splenic IFN-γ^+^ or IL-4^+^ T cell frequencies and heart Th1/Th2 cytokine profiles were respectively evaluated by FACS and ELISA on day 8 following CVB3 infection. As shown in Fig. 5A, 3.79% of CD4^+^IFN-γ^+^ and 3.85% CD8^+^IFN-γ^+^ T cells were evidenced in pIP-10-AT-treated mice, significantly lower than those of pcDNA3.1-treated mice (CD4^+^IFN-γ^+^ T cells: 5.27%; CD8^+^IFN-γ^+^ T cells: 6.01%, *p*<0.05). While, percentages of CD4^+^/CD8^+^ IL-4^+^ T cells did not showed remarkable differences between these two groups. Similarly, the levels of cardiac Th1 cytokines (IFN-γ, IL-12, TNF-α) were extensively and dramatically decreased in pIP-10-AT treated mice compared with other groups. As high as 960 pg/ml of IFN-γ and 1150 pg/ml of TNF-α were reduced to lower levels (510 pg/ml and 330 pg/ml). In contrast, hardly changed Th2 cytokine expressions were noticed except of the slight down-regulation of IL-13, indicating that pIP-10-AT treatment efficiently impaired Th1 immune responses by significantly reducing Th1 cytokine production, which may ameliorate the CVB3-induced myocardial injury.

## Discussion

Viral myocarditis is an inflammation of the myocardium that follows enterovirus or adenovirus infections. It is the composite result of both virus infection and host uncontrolled immune reactions [20]. During the early viremia period, CVB3 infects heart tissues by receptor-mediated endocytosis [21] and may cause myocardiocyte dysfunction by disrupting dystrophin-sarcoglycan complex or cleaving eukaryotic initiation factor-4 [22]. At later stage of infection, proinflammatory cytokines and chemokines are robustly expressed and result in the massive inflammation and aggravated injury in heart [23]. Our previous study demonstrated that cardiac MCP-1, a Th1 chemokine, was obviously up-regulated *in vivo* and *in vitro* following CVB3 infection and was one of the leading causes of the cardiac inflammation and immune injury [8]. Other reports indicate that the expression pattern, level and kinetics of cytokines, chemokines and their receptors are closely related to the progress and severity of inflammatory heart diseases [24]–[27]. Therefore to re-establish an immune balance by modulating chemokine and cytokine expressions represents as a potential therapeutic strategy for viral myocarditis.

Recent studies showed that chemokine IP-10 was induced in heart tissue following CVB3 infection [13], [28]. We confirmed this phenomenon in this study and for the first time extensively described the expression kinetics of cardiac IP-10 in CVB3 infected mice. We found that IP-10 was induced as early as day 1 post-infection, peaked at day 4, and then slightly decayed and maintained a high level until day 7, appearing in a time-dependent manner. This expression pattern was coincident with the kinetic changes of cardiac CVB3 titers, which also peaked at day 3–4 and then gradually subsided [29]. Meanwhile, the cardiac IP-10 expression also increased in parallel with the virus dose. These consistencies reflect the intimate interaction and communication between host immunity and CVB3 virus, and may indicate that the IP-10 induction is the host essential immune response for CVB3 infection. Weinzierl AO et al [13] reported the benefit role of IP-10 at the initial stage of CVB3 infection using IP-10^−/−^ mice model. Yuan J et al [28] recently demonstrated that IP-10 helped to inhibit viral replication at the early stage of CVB3 myocarditis (at day 3 post-infection). However, at the climax of acute infection (day 7 to 10 post-infection), neither over-expression nor deficiency of IP-10 could significantly alter the viral clearance, myocarditis severity or mouse survival rate, suggesting an early and temporal virus-limiting effect and a complex role of IP-10 in the course of CVB3 myocarditis.

It is well-established that in the sub-acute stage of CVB3 myocarditis (day 4–14), excessive immune responses become the dominant damage factor instead of virus virulence [30]. Th1-dominant immunity has been considered as one of the important mechanisms in the development of CVB3 myocarditis, and the shift of Th1 to Th2 immune response could alleviate the myocarditis severity [31]. IP-10 is considered as a Th1-type chemokine and plays a critical role in many Th1-mediated diseases, such as colitis [32], sarcoidosis [33], inflammatory lung injury [34] and visceral leishmaniasis [35]. Thus, blocking IP-10/CXCR3 signaling pathway may significantly impair the induction and recruitment of Th1 cells to local tissue site, weaken the secondary tissue injury and improve organ functions.

In this study, a plasmid encoding mutant IP-10 (pIP-10-AT), which antagonize IP-10-mediated cell chemotaxis by competitively binding CXCR3 receptor (Fig. 3B), was used to treat mice after CVB3 infection. We found that pIP-10-AT injection significantly improved the pancreas injury as well as myocarditis manifestations including serum indices and myocardial histopathology. These protective roles were not due to the control of viral replication since no decline of cardiac or pancreatic viral titer was evidenced, but rather partially resulted from the efficient blockade of the inflammatory cell trafficking into tissues, as demonstrated in Figs. 3C and 4H. Blocking endogenous IP-10 also led to a significant rectification of Th1 dominant immune responses, evidenced by decreased frequencies of splenic CD4^+^IFN-γ^+^ and CD8^+^IFN-γ^+^ T cells, reduced cardiac Th1 cytokine levels (IFN-γ, IL-12 and TNF-α), and hardly no decreased Th2 cytokine expressions except of IL-13. IL-13 has been shown to exert protective effects against CVB3 myocarditis by suppressing proinflammatory cytokine production and down-regulating T-cell immune responses [36]. The inconsistency between the decreased protective IL-13 and the improved myocarditis by pIP-10-AT might be explained by an overwhelming beneficial effect of Th1 cytokine reduction to a relevant weak detrimental effect of solely Th2 cytokine (IL-13) decrease.

In conclusion, we report for the first time the protective effect of IP-10 blocking strategy in treating CVB3-induced myocarditis, and demonstrate the pIP-10-AT injection as an effective means to antagonize endogenous IP-10 function, and thus suppress chemokine-directed immune inflammation and resultant myocardial injury, when therapy was applied after the onset of disease. This IP-10-antagonism strategy may provide an alternative therapeutic approach in the future treatment of viral myocarditis and possible other Th1-mediated inflammatory heart diseases.

## Materials and Methods

### Ethics Statement

This study was carried out in strict accordance with the recommendations in the Guide for the Care and Use of Medical Laboratory Animals (Ministry of Health, P. R. China, 1998). The protocol was approved by the Medical Laboratory Animal Care and Use Committee of Jiangsu Province (Permit Number: SYXK 2007-0035) as well as the Ethical Committee of Soochow University (Permit Number: 2007011).

### Mice and virus

Male inbred BALB/c (H-2^d^) mice 6–8 weeks of age were purchased from the Experimental Animal Center of Chinese Academy of Science (Shanghai, P. R. China). CVB3 (Nancy strain) was a gift from Professor Yingzhen Yang (Key Laboratory of Viral Heart Diseases, Zhongshan Hospital, Shanghai Medical college of Fudan University) and was maintained by passage through Hela cells (ATCC number: CCL-2). Viral titer was routinely determined prior to infection by a 50% tissue culture infectious dose (TCID_50_) assay of Hela cell monolayer.

### Cardiac IP-10 ELISA assay

Freshly isolated heart tissues were ground to a fine powder under liquid nitrogen. Each sample (100 mg of powdered tissue) was homogenized in 0.5 ml RIPA buffer and subsequently centrifuged in a cooled micro-centrifuge for 8 min. The supernatants were then subjected to ELISA assays with mouse IP-10 construction ELISA kits (Antigenix America Inc).

### Construction of pIP-10-AT plasmid

Plasmid encoding murine IP-10 (pIP-10) has been constructed previously [37]. The IP-10-AT (AT: antagonist) gene with a 6×His tag at the C-terminal was yielded by method of gene splicing to add a methionine after removal of the first 5 N-terminal amino acids. Following confirmation of the entire nucleotide sequence, the fragment was then incorporated into the eukaryotic expression plasmid pcDNA3.1.

### Expression of pIP-10-AT plasmid

Human embryonic kidney 293T cells (ATCC CRL-11268) were transfected with pIP-10 or pIP-10-AT by lipofectamine (Invitrogen) and cultured for 48 h. The supernatants of transfected cells were collected and subjected to mouse IP-10 quantitative ELISA assay according to manufacturer's instructions (Antigenix America Inc).

### Chemotaxis assays *in vitro* and *in vivo*

The chemotaxis assay *in vitro* was conducted using a modified 48-well Boyden chamber migration assay. Concanavalin A (ConA) activated splenocytes were transferred to upper chambers (1×10^5^ cells/50 µl). The supernatant from pIP-10-AT or pIP-10 transfected 293T cells was pretreated with anti-IP-10 (10 µg/ml) or isotype antibody before adding to the lower chamber. IP-10 protein prepared by ourselves was used as a positive control. After 4 h incubation, migration was expressed as cell number per five high-power fields in the polycarbonate chemotaxis membrane, with duplicate wells being counted for each of three experiments. The chemotaxis ability of pIP-10 or pIP-10-AT was evaluated by the chemotactic index [(number of cells migrating in experimental well)/(number of cells migrating in medium well)].

The chemotaxis assay *in vivo* was conducted as follows: mice were injected with 100 µg pIP-10-AT, 100 µg pIP-10, 100 µg pIP-10-AT plus 100 µg pIP-10 or 100 µg pcDNA3.1 at the sites of femoral muscle. 3 days later, the muscle tissues were fixed in 10% buffered formalin, sectioned and stained with hematoxylin and eosin (HE). Sections were observed using a Nikon Eclipse TE2000-S microscope with magnification ×200, and evaluated for the blockade effect of pIP-10-AT on pIP-10-mediated chamotaxis.

### Competitive binding assay

The competitive binding ability of IP-10-AT with IP-10 to their receptor CXCR3 was detected as described previously [38]. Recombinant IP-10 and IP-10-AT proteins were expressed in E. coli and then purified by the methods discribed previously [39]. Mouse CXCR3 stable-transfected CHO cells (ATCC CCL-61, 4×10^5^) were incubated with FITC-labeled IP-10 (20 µg/ml) with or without unlabeled IP-10-AT or BSA (200–600 µg/ml) for 1 hour at 4°C and washed extensively to remove unbound protein, and analyzed by flow cytometry.

### CVB3 infection and pIP-10-AT therapy

Mice were intraperitoneally infected with 10^3^ TCID_50_ CVB3 at day 0, and then immediately received intramuscular injection of 100 µg pIP-10-AT twice at day 0 and day 3. Mice receiving pcDNA3.1 were used as a negative control group.

### Tissue histopathology and myocarditis grading

8 days following CVB3 infection, heart and pancreas tissues of mice were collected, sectioned and subjected to HE staining. Heart sections were examined by two independent investigators in a blinded manner, and the severity of myocarditis was assessed by a previously described 0–4 scale [40], in which 0 =  no inflammation; 1 =  one to five distinct mononuclear inflammatory foci with involvement of 5% or less of the cross-sectional area; 2 =  more than five distinct mononuclear inflammatory foci, or involvement of over 5% but not over 20% of the cross-sectional area; 3 =  diffuse mononuclear inflammation involving over 20% of the area, without necrosis; and 4 =  diffuse inflammation with necrosis.

### Intracellular staining

8 days following CVB3 infection, splenocytes (2×10^6^/ml) were isolated and reactivated *in vitro* with 10 µg/ml of plate-bound anti-CD3 (eBioscience) and 2 µg/ml soluble anti-CD28 antibody (eBioscience) for 24 h. For the final 4 h, 1 µg/ml of GolgiStop (BD Pharmingen) was added. Cells were harvested and stained with FITC-conjugated anti-mouse CD4 or anti-mouse CD8 antibody (eBioscience), after washing the cells were fixed with fixation buffer (eBioscience) for 20 min at 4°C and permeabilized with permeabilization solution (eBioscience) for 20 min at room temperature, and then stained with PE-conjugated anti-mouse IFN-γ or anti-mouse IL-4 (eBioscience). The percentage of cytokine-secreting CD4^+^ or CD8^+^T cells was determined by flow cytometry using a FACScalibur instrument (Becton Dickinson).

### Cytokine ELISA

Expressions of IFN-γ, IL-12, TNF-α, IL-4, IL-10 and IL-13 in the homogenized heart tissues were measured by ELISA assays (R&D, except IL-13 ELISA kit from eBioscience) following the manufacturers' instructions. In brief, plates were coated with diluted capture antibody (100 µl/well) and incubated overnight at 4°C. Samples and standards were added to triplicate wells and incubated at 37°C for 2 h. After washing, biotinylated detection antibody was added for 1 h, followed by 100 µl strepavidin conjugated horseradish peroxidase for 1 h at 37°C. TMB substrate (eBioscience) was added to each well. After 10 min, 50 µl of stop solution (2 N H_2_SO_4_) was added and absorbance was measured at 450 nm.

### Quantization of tissue viral load

8 days after 10^3^ TCID_50_ CVB3 infections, heart and pancreas tissues were collected, weighed and frozen at -70°C in 10% FBS-RPMI 1640 medium. Samples were later thawed, homogenized, serially diluted in 10-fold increments, and incubated on confluent Hela cell monolayer for 1 h at 37°C and 5% CO_2_ to allow virus attachment, and then incubated for 7 days to allow plaque formation. Viral titer was expressed as the mean PFU/100 mg tissue ± SD.

### Statistical analysis

Data are shown as the mean ± SD. Statistical analysis of the data was performed using the GraphPad Prism (Version 4.0) statistical program. Means were compared using the Student's two-sample t-test. The survival rates of CVB3 infected mice were compared and analyzed with Kaplan-Meier plot. *p*<0.05 was considered statistically significant.
